# Role of Salivary Uric Acid Versus Serum Uric Acid in Predicting Maternal Complications of Pre-Eclampsia in a Rural Hospital in Central India: A Two-Year, Cross-Sectional Study

**DOI:** 10.7759/cureus.23360

**Published:** 2022-03-21

**Authors:** Sparsh Madaan, Arpita Jaiswal, Neema Acharya, Surekha Tayade, Archana Dhok, Sunil Kumar, Sourya Acharya, Deepika Dewani, Dhruv Talwar, Dhruva Halani, Manila Reddy Eleti

**Affiliations:** 1 Department of Obstetrics and Gynaecology, Jawaharlal Nehru Medical College, Datta Meghe Institute of Medical Sciences (Deemed to be University), Wardha, IND; 2 Department of Biochemistry, Jawaharlal Nehru Medical College, Datta Meghe Institute of Medical Sciences (Deemed to be University), Wardha, IND; 3 Department of Medicine, Jawaharlal Nehru Medical College, Datta Meghe Institute of Medical Sciences (Deemed to be University), Wardha, IND

**Keywords:** socioeconomic status, maternal complications, pre-eclampsia, serum uric acid, salivary uric acid

## Abstract

Background

Hypertensive disorders during pregnancy are an important topic of concern, specifically in rural and remote areas of India where there is a lack of awareness and it is difficult to maintain proper follow-up of pregnant females to screen them for complications developed during pregnancy. Gestational hypertension and pre-eclampsia result in the abruption of the placenta, hemolysis, elevated liver enzymes, low platelet count syndrome, eclampsia, and disseminated intravascular coagulation, which can be a serious threat to the health of the mother and the fetus. Therefore, it is important to identify biomarkers for diagnosing and predicting the complications of pre-eclampsia that may aid the obstetric high-dependency units based in rural areas to tackle this important health hazard during pregnancy.

Methodology

A total of 180 singleton pregnant women of more than 34 weeks of gestational age were enrolled in this study. All women were divided into three groups (control group, severe pre-eclampsia, and non-severe pre-eclampsia) based on the severity of blood pressure and the presence of proteinuria (≥+1 by the dipstick method). Salivary and serum uric acid levels were measured through morning samples, and all patients were monitored for the development of complications and outcomes. Salivary uric acid and serum uric acid levels were correlated with each other and with maternal complications of pre-eclampsia.

Results

Mean salivary uric acid (mg/dL) in severe pre-eclampsia was (6.72 ± 0.49) significantly higher compared to non-severe pre-eclampsia (4.75 ± 0.94) and control (3.13 ± 0.43). Mean serum uric acid (mg/dL) in severe pre-eclampsia was (8.13 ± 0.87) significantly higher compared to non-severe pre-eclampsia (6.23 ± 0.76) and control (3.85 ± 0.46).The lowest best cut-off value of maternal salivary uric acid was 5.06 mg/dL, above which one can predict maternal complications with a diagnostic accuracy of 78.33%.

Conclusions

Salivary uric acid and serum uric acid levels are significantly raised in cases of pre-eclampsia in comparison to normal pregnancy. Salivary uric acid and serum uric acid are correlated significantly indicating that salivary uric acid can function as a cost-effective, novel marker to provide an idea about serum uric acid levels. The prognostic accuracy of salivary uric acid was good in predicting maternal complications among cases of pre-eclampsia (severe and non-servere) and early-onset maternal complications. Therefore, it may be utilized as a helpful marker to identify high-risk patients.

## Introduction

Hypertensive disorders of pregnancy (HDP) are common complications during pregnancy with reported incidence rates ranging from approximately 2% to 8% [1,2]. These disorders can negatively impact gestational outcomes. One of the most deleterious gestational hypertensive disorders is pre-eclampsia, which is characterized by gestational hypertension and nephrotic impairment with proteinuria [1]. Pre-eclampsia is associated with increased mortality rates during pregnancy [1,2].

The prevalence of HDP, gestational hypertension, and pre-eclampsia is 5.2-8.2%, 1.8-4.4%, and 0.2-9.2%, respectively, [3-5]. The prevalence of maternal and fetal complications associated with HDP varies by region and healthcare facility type [3]. HDP has been reported to cause approximately 60-80% of all maternal deaths [3]. The American High Blood Pressure Education Program Working Group report indicated that about 30% of HDP in America were caused by chronic hypertension, while 70% of the cases were caused by pre-eclampsia [3]. Hyperuricemia is a common finding in pregnant women with pre-eclampsia that often precedes hypertension and proteinuria [5]. There are several potential origins for uric acid in pre-eclampsia, such as abnormal renal function, increased tissue breakdown, acidosis, and increased activity of the xanthine oxidase/dehydrogenase enzyme.

The importance of uric acid lies in association with pre-eclampsia because it is associated with the severity of pre-eclampsia and adverse fetal outcomes as reported by previous studies [3-10]. In addition to estimation of serum uric acid levels, previous studies have highlighted the presence of uric acid in saliva [3,4]. Further, studies have shown a linear relationship between serum and saliva uric acid levels, and thus, saliva can serve as a useful surrogate marker for blood testing in uric acid estimation among pre-eclamptic women.

Because saliva collection is easy, non-invasive, and cost-effective requiring simple instructions for collection, salivary uric acid testing can be a useful approach for monitoring pre-eclampsia at home and hospital settings instead of lab-based serum uric acid testing.

## Materials and methods

This cross-sectional study conducted from September 2019 to August 2021 enrolled a total of 180 women having singleton pregnancies with ≥34 weeks of gestational age. The study site was a rural-based tertiary care institute catering to around 2,000 deliveries per year. The incidence of pre-eclampsia in the geographic region of the study site ranges from 9% to 15% [11]. Considering the incidence of 12% and using the value as 1.96 at 5% type I error, the sample size was calculated using the following formula: $(〖Z_(1-&prop;/2)〗^2 p(1-p))/d^2$, where p is the expected proportion in population based on previous studies or the pilot study as 10% = 0.1, 1-p = 1-0.1 = 0.9, d is the absolute error or precision of 5% = 0.05.

$N= (〖1.96〗^2 x0.12 (1-0.12))/(0.05)=163$

**Figure 1:**
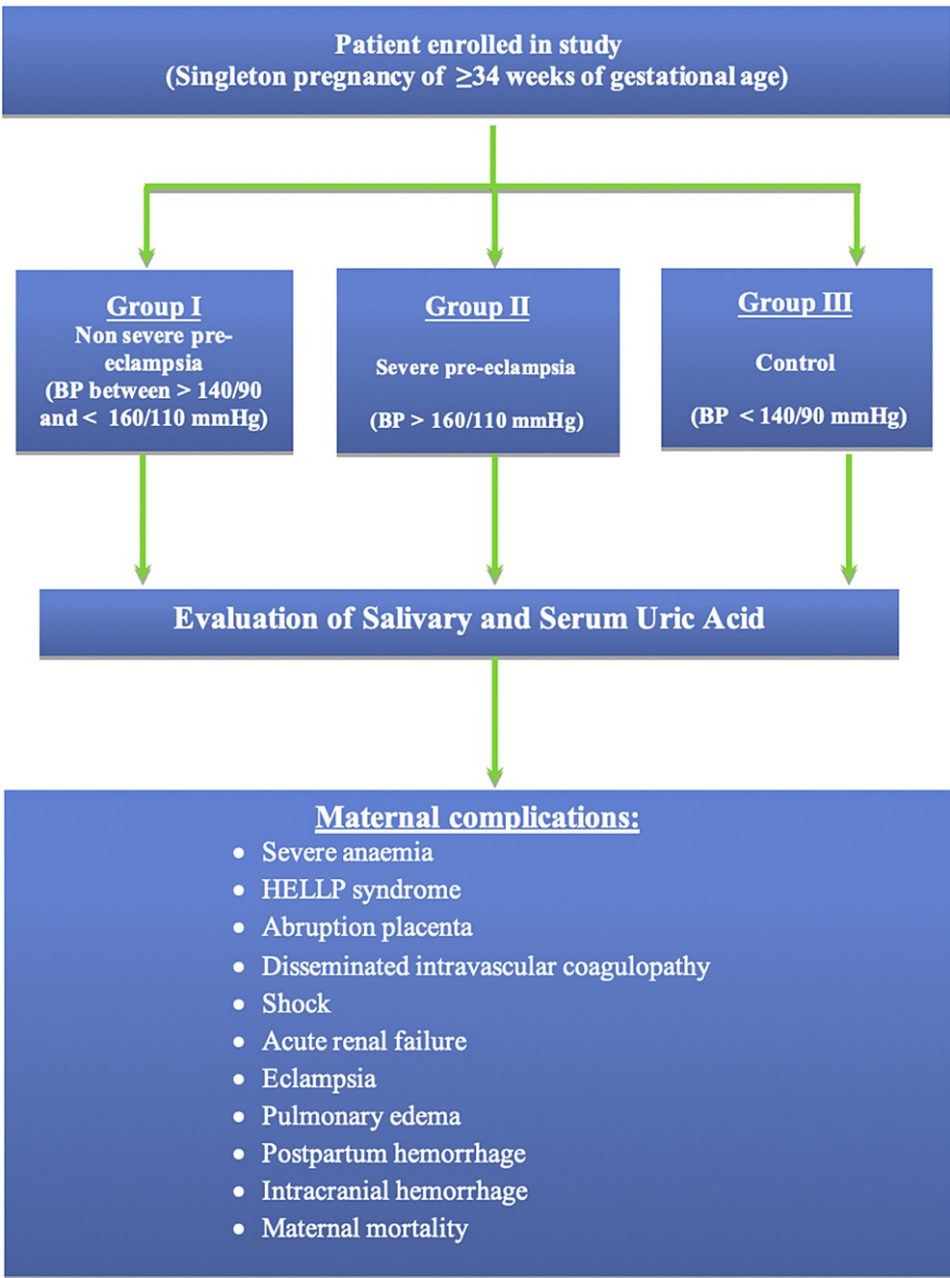
Study methodology. BP: blood pressure; HELLP: hemolysis, elevated liver enzymes, low platelet count

We included singleton pregnant women with pre-eclampsia and ≥34 weeks of gestational age. The criteria for the diagnosis of pre-eclampsia included a blood pressure of ≥140/90 mmHg on two occasions four hours apart with or without proteinuria 30 mg/dL (≥1+ dipstick test) in random urine samples. Exclusion criteria included multiple pregnancies, diabetes mellitus, heart disease, gout, autoimmune disorder, connective tissue disorder, renal and liver disease, and a history of thromboembolism. The study methodology is shown in Figure 1.

All patients were divided into three groups according to their blood pressure and proteinuria. The study group I (n = 60) comprised non-severe pre-eclampsia where the blood pressure was between ≥140/90 and <160/110 mmHg, with or without proteinuria. The study group II (n = 60) comprised severe pre-eclampsia where the blood pressure was ≥160/110 mmHg with or without proteinuria. The study group III (n = 60) comprised control where the blood pressure was <140/90 mmHg with or without proteinuria.

The sociodemographic data including age, residence, education, occupation, and socioeconomic status (evaluated by the modified B. G. Prasad scale) were noted [12]. A predesigned, pretested proforma was used to collect the sociodemographic information, clinical history, clinical examination findings, and investigation status results.

The complete history of present pregnancy was noted, and general examination (including blood pressure measured in the right arm in sitting position, maternal pulse, temperature, pallor, edema) was performed. On admission for delivery, an obstetric examination was done. On recruitment for the study, serum uric acid and salivary uric acid levels were measured quantitatively.

After delivery, data were collected on the mode of delivery and maternal outcomes. Maternal complications that arose during the antenatal period and delivery were recorded in terms of severe anemia, HELLP syndrome, placental abruption, disseminated intravascular coagulopathy, shock, acute renal failure, eclampsia, pulmonary edema, postpartum hemorrhage, and intracranial hemorrhage.

Salivary uric acid

Unstimulated whole expectorated saliva from each pregnant woman was collected into sterile tubes at 8-9 AM after rinsing of the mouth with 5 mL of distilled water to wash out exfoliated cells. Samples were stored in a refrigerator at 4°C and centrifuged. The supernatant was used for the analysis of uric acid. Estimation of salivary uric acid was done using the colorimetric method on an automated clinical chemistry analyzer (VITROS 5600, Ortho Clinical Diagnostics, Raritan, NJ, USA).

Serum uric acid

A 2 mL venous blood sample was collected at the same time to measure serum uric acid levels. Samples were centrifuged and the supernatant was used for the analysis of uric acid. Estimation of serum uric acid was done using the colorimetric method on an automated clinical chemistry analyzer (VITROS 5600, Ortho Clinical Diagnostics, Raritan, NJ, USA).

**Figure 2:**
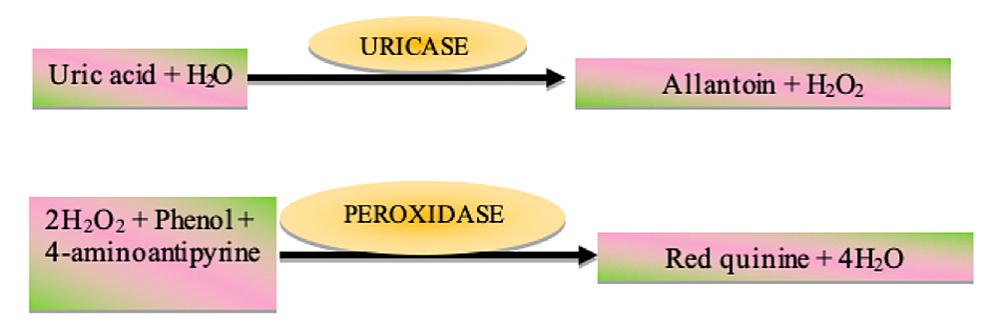
The principle of the modified uricase-PAP method.

Both serum and salivary uric acid levels were estimated using the modified uricase-PAP method (Figure 2). An enzyme uricase converts uric acid into allantoin and hydrogen peroxide. Hydrogen peroxide reacts with phenol and 4-amino antipyrine in the presence of the peroxidase enzyme to form a red-colored quinoneimine dye complex. The intensity of the color developed is directly proportional to the amount of uric acid present in the sample.

Study definitions

Sociodemographic factors included age, education expressed as years of schooling, and socioeconomic status. Regarding education level, an illiterate was defined as a person aged more than seven years who could neither read nor write or those who could merely read but could not write in any language. A person who studied from class first to seventh was classified as having primary education, whereas those who studied from class eighth to twelfth were classified as having secondary education. A person undergoing or completing a graduation course or above was classified as a graduate or above [13]. Patients were classified according to their per-capita income using the Modified B. G. Prasad classification (Table 1) [12].

**Table 1:** The modified B. G. Prasad classification.

Social class	Original classification of per-capita income (INR/month)	Revised classification for 2021 (INR/month)
Upper class	100 and above	7,770 and above
Upper-middle class	50–99	3,808–7,769
Middle class	30–49	2,253–3,808
Lower-middle class	15–29	1,166–2,253
Lower class	<15	<1,166

Weight (in kilograms) was measured on a single, digital weighing machine and recorded to the nearest 100 g. Height (in meters) was recorded using a stadiometer.

The criteria adopted for the diagnosis of hypertension was from the Working Group Classification of the National High Blood Pressure Education Programme (2000) [10]. Gestational hypertension was defined as the systolic blood pressure of >140 mmHg, or diastolic blood pressure of >90 mmHg, for the first time in pregnancy after 20 weeks of gestation, without proteinuria, with blood pressure returning to normal before 12 weeks postpartum [14].

Pre-eclampsia was defined as systolic blood pressure of >140 mmHg or diastolic blood pressure of >90 mmHg after 20 weeks of gestation and proteinuria of >300 mg/24 hours or >1 + dipstick or systemic complications. Mild pre-eclampsia was defined as systolic blood pressure of >140 but <160 mmHg or diastolic blood pressure of >90 mmHg but <110 mmHg after 20 weeks of gestation with proteinuria >300 mg but <5 g/24 hours or dipstick >1 + but <3 +, without any systemic complications. Severe pre-eclampsia occurred when blood pressure crossed 160/110 mmHg with proteinuria 25 g/24 hours or 3 + dipstick, along with systemic signs of end-organ disease such as headache, visual disturbances, abdominal thrombocytopenia, pulmonary edema, fetal growth restriction, pain, and oliguria. Seizures that could not be attributed to other causes in women with pre-eclampsia were attributed to eclampsia.

Anemia was defined as hemoglobin below 11 g/dL and hematocrit of less than 33%. According to the classification provided by the Indian Council of Medical Research, mild anemia was defined by a hemoglobin level of 10.0-10.9 g/dL for pregnant women. Moderate anemia was defined by a hemoglobulin level of 7.0-9.9 g/dL, severe anemia corresponded to a level less than 7.0 g/dL, and very severe anemia corresponded to a level less than 4.0 g/dL [15].

Pallor is the pale color of the skin that can be caused by illness, emotional shock or stress, stimulant use, or anemia. Pallor is the result of a reduced amount of oxyhemoglobin and can be visible as pallor of the conjunctivae of the eyes on physical examination [16]. Mild pallor was defined as pallor of the conjunctiva and/or mucous membrane. Moderate pallor was defined as pallor of the conjunctiva and/or mucous membrane along with pallor of the skin. Severe pallor was defined as pallor of the conjunctiva and/or mucous membrane with the pallor of the skin, along with the pallor of palmar creases.

Edema is the observable swelling due to fluid accumulation in body tissues. Edema is classified into two types, namely, pitting and non-pitting edema. Pitting edema responds to pressure either from a finger or a hand while pitting edema does not. Pitting edema is further classified based on the depth and duration of the indentation. The following scale was used to rate the severity [17]: Grade 1 was defined as the pressure applied by the doctor leaving an indentation of 0-2 mm that rebounded immediately. Grade 2 was defined as the pressure leaving an indentation of 3-4 mm that rebounded in fewer than 15 seconds. Grade 3 was defined as pressure leaving an indentation of 5-6 mm that took up to 30 seconds to rebound. Grade 4 was defined as the pressure leaving an indentation of 8 mm or deeper and taking more than 20 seconds to rebound.

Maternal complications included a new episode of hypertension with blood pressure (≥160/110 mmHg [18]), renal insufficiency, increased liver enzymes, signs of neurological involvement, presence of thrombocytopenia, HELLP syndrome, gestational diabetes mellitus, placental abruption, disseminated intravascular coagulopathy, shock, acute renal failure, pulmonary edema, intracranial hemorrhage, and maternal mortality. Renal insufficiency was defined as serum creatinine of >1.2 mg/dL or oliguria (<400 mg/dL) [19]. Increased liver enzymes were defined as aspartate aminotransferase of >40 U/L or alanine aminotransferase above 50 U/L [20]. Signs of neurological involvement included hyperreflexia or clonus, wherein hyperreflexia was an exaggerated response of the deep tendon reflexes, usually resulting from injury to the central nervous system or metabolic disease [21], and clonus was involuntary and rhythmic muscle contractions caused by a permanent lesion in descending motor neurons [22]. Thrombocytopenia was defined as a platelet count of <150 × 10^9^/L [18]. HELLP syndrome was defined as hemolysis (H), elevated liver enzymes (EL), and low platelet count (<100,000/mm^3^) [23]. Gestational diabetes mellitus was defined as carbohydrate intolerance of variable severity with onset or first recognition during the present pregnancy. Gestational diabetes mellitus was diagnosed at any time in pregnancy if one or more of the following criteria was met [24,25]: Fasting plasma glucose level of >126 mg/dL (7.0 mmol/L), a random blood glucose level of >200 mg/dL (11.1 mmol/L), or HbA1c of ≥5.9%. Abruptio placenta was defined as a hemorrhage wherein the bleeding occurred due to premature separation of normally situated placenta [26]. Disseminated intravascular coagulopathy was defined as a widespread hypercoagulable state that could lead to both microvascular and macrovascular clotting and compromised blood flow, ultimately resulting in multiple organ dysfunction syndromes with low platelet count, elevated D-dimer concentration, decreased fibrinogen concentration, and prolongation of prothrombin time [27]. Shock was defined as a state of cellular and tissue hypoxia due to either reduced oxygen delivery, increased oxygen consumption, inadequate oxygen utilization, or a combination of these processes [28]. Acute renal failure was defined as a rapid fall in the rate of glomerular filtration, which manifested clinically as an abrupt and sustained increase in the serum levels of urea and creatinine with an associated disruption of salt and water homeostasis [29]. Pulmonary edema was defined as a buildup of fluid in the alveoli (air spaces) of the lungs [30]. Intracranial hemorrhage was defined as bleeding within the intracranial vault [31]. Maternal mortality was defined according to the World Health Organization as the death of a woman from pregnancy-related causes during pregnancy or within 42 days of pregnancy, expressed as a ratio of 100,000 live births in the population being studied [32].

Statistical analysis

Categorical variables were presented in the form of numbers and percentages. On the other hand, quantitative data with normal distribution were presented as the mean ± standard deviation (SD) and as median with 25th and 75th percentiles (interquartile range). The data normality was checked using the Kolmogorov-Smirnov test. For cases in which the data was not normal, we used non-parametric tests. The comparison of non-normally distributed quantitative data was analyzed using the Mann-Whitney test (for two groups) and the Kruskal Wallis test (for more than two groups), and post hoc comparison was done using Dunn’s multiple pairwise comparison test. The comparison of the qualitative variables was analyzed using the chi-square test. If any cell had an expected value of less than 5, then Fisher’s exact test was used. Spearman rank correlation coefficient was used to correlate salivary uric acid (mg/dL) and serum uric acid (mg/dL).

The data entry was done in a Microsoft Excel spreadsheet, and the final analysis was done using SPSS version 21.0 (IBM Corp., Armonk, NY, USA). The receiver operating characteristic curve was used to find the cut-off point of salivary uric acid and serum uric acid for predicting complications. For statistical significance, p-values of less than 0.05 were considered statistically significant.

Ethical approval

Ethical approval for this study was obtained from the Institutional Ethical Committee with the corresponding approval number: DMIMS (DU)/IEC/Sept-19/8396 on May 10, 2019. Informed consent was obtained from all study participants before their enrolment in the study.

## Results

Out of the 180 women, 140 (77.78%) were in the age group of 21-30 years, 29 (16.11%) women were in the age group of >30 years, and 11 (6.11%) women were in the age group of <19 years. The distribution and correlation of different parameters among the three groups are presented in Tables 2, 3.

**Table 2:** Distribution and correlation of different parameters among the three study groups.

Parameter	Severe pre-eclampsia (n = 60)	Non-severe pre-eclampsia (n = 60)	Control (n = 60)	Total	P-value
Maternal age (years)					
Mean ± SD	26.37 ± 4.67	26.35 ± 4.41	26.42 ± 4.22	26.38 ± 4.41	0.942 (not significant)
Range	19–45	20–39	19–39	19–45	
Socioeconomic status					
Lower class	22 (36.67%)	11 (18.33%)	5 (8.33%)	38 (21.11%)	<0.0001 (significant)
Lower-middle class	19 (31.67%)	20 (33.33%)	9 (15%)	48 (26.67%)	
Middle class	11 (18.33%)	15 (25%)	13 (21.67%)	39 (21.67%)	
Upper-middle class	6 (10%)	10 (16.67%)	21 (35%)	37 (20.56%)	
Upper class	2 (3.33%)	4 (6.67%)	12 (20%)	18 (10%)	
Total	60 (100%)	60 (100%)	60 (100%)	180 (100%)	
Gravidity					
Primigravida	24 (40%)	22 (36.67%)	25 (41.67%)	71 (39.44%)	0.85 (not significant)
Multigravida	36 (60%)	38 (63.33%)	35 (58.33%)	109 (60.56%)	
Total	60 (100%)	60 (100%)	60 (100%)	180 (100%)	

**Table 3:** Correlation of gestational age, salivary, and serum uric acid in controls, pre-eclampsia, and non-severe pre-eclampsia patients.

Parameter	Severe pre-eclampsia (n = 60)	Non-severe pre-eclampsia (n = 60)	Control (n = 60)	P-value
Gestational age (weeks)				
Mean ± SD	38.47 ± 0.98	38.28 ± 1.13	38.36 ± 1.15	0.693 (not significant)
Range	37–41.1	35.4–41.1	35.2–41.1	
Salivary uric acid				
Mean ± SD	6.72 ± 0.49	4.75 ± 0.94	3.13 ± 0.43	<0.0001 (significant)
Range	5.82–7.6	3.06–6.8	2.09–4.6	
Serum uric acid				
Mean ± SD	8.13 ± 0.87	6.23 ± 0.76	3.85 ± 0.46	<0.0001 (significant)
Range	6.2–9.41	5.02–7.49	2.99–5	

Serum and salivary uric acid levels were significantly associated with the mode of delivery in severe and non-severe pre-eclampsia groups, as shown in Table 4. Serum and salivary uric acid levels were significantly raised in patients undergoing lower segment cesarean section (LSCS) versus vaginal delivery. However, no significant association of uric acid levels was found with the mode of delivery in the control group.

**Table 4:** Association of uric acid levels with the mode of delivery in severe pre-eclampsia, non-severe pre-eclampsia, and control patients. LSCS: lower segment cesarean section

	Uric acid (mg/dL)	Vaginal delivery	LSCS	Total	P-value
Severe pre-eclampsia	Salivary uric acid (mg/dL)				
	Mean ± SD	6.15 ± 0.29	6.85 ± 0.44	6.72 ± 0.49	<0.0001 (significant)
	Range	5.82–6.7	5.82–7.6	5.82–7.6	
	Serum uric acid (mg/dL)				
	Mean ± SD	7.11 ± 0.4	8.35 ± 0.79	8.13 ± 0.87	<0.0001 (significant)
	Range	6.2-7.6	6.2-9.41	6.2-9.41	
Non-severe pre-eclampsia	Salivary uric acid (mg/dL)				
	Mean ± SD	4.1 ± 0.75	4.98 ± 0.89	4.75 ± 0.94	0.0008 (significant)
	Range	3.12–5.88	3.06–6.8	3.06–6.8	
	Serum uric acid (mg/dL)				
	Mean ± SD	5.71 ± 0.51	6.42 ± 0.75	6.23 ± 0.76	0.0005 (significant)
	Range	5.08–6.8	5.02–7.49	5.02–7.49	
Control	Salivary uric acid (mg/dL)				
	Mean ± SD	3.03 ± 0.32	3.2 ± 0.49	3.13 ± 0.43	0.212 (not significant)
	Range	2.09–3.8	2.09–4.6	2.09–4.6	
	Serum uric acid (mg/dL)				
	Mean ± SD	3.82 ± 0.47	3.88 ± 0.47	3.85 ± 0.46	0.626 (not significant)
	Range	2.99–4.52	2.99–5	2.99–5	

As shown in Table 5, both serum and salivary uric acid levels were significantly associated with the type of LSCS in severe and non-severe pre-eclampsia groups. Serum and salivary uric acid levels were significantly raised in patients undergoing emergency LSCS versus elective LSCS. However, no significant association of uric acid levels was found with the type of LSCS in the control group.

**Table 5:** Association of uric acid levels with the type of lower segment cesarean section in severe pre-eclampsia, non-severe pre-eclampsia, and control patients.

	Uric acid (mg/dL)	Elective	Emergency	Total	P-value
Severe pre-eclampsia	Salivary uric acid (mg/dL)				
	Mean ± SD	6.31 ± 0.47	6.95 ± 0.35	6.85 ± 0.44	<0.0001 (significant)
	Range	5.82–7.2	6.09–7.6	5.82–7.6	
	Serum uric acid (mg/dL)				
	Mean ± SD	7.23 ± 0.55	8.57 ± 0.62	8.35 ± 0.79	<0.0001 (significant)
	Range	6.2–8.06	7.02–9.41	6.2–9.41	
Non-severe pre-eclampsia	Salivary uric acid (mg/dL)				
	Mean ± SD	4.11 ± 0.65	5.49 ± 0.55	4.98 ± 0.89	<0.0001 (significant)
	Range	3.06–5.09	4.99–6.8	3.06–6.8	
	Serum uric acid (mg/dL)				
	Mean ± SD	5.6 ± 0.44	6.89 ± 0.4	6.42 ± 0.75	<0.0001 (significant)
	Range	5.02–6.2	6.22–7.49	5.02–7.49	
Control	Salivary uric acid (mg/dL)				
	Mean ± SD	3.15 ± 0.46	3.4 ± 0.63	3.2 ± 0.49	0.469 (not significant)
	Range	2.09–4.6	2.89–4.6	2.09–4.6	
	Serum uric acid (mg/dL)				
	Mean ± SD	3.82 ± 0.46	4.14 ± 0.44	3.88 ± 0.47	0.303 (not significant)
	Range	2.99–5	3.8–5	2.99–5	

As shown in Table 6, the proportion of patients with neonatal intensive care unit (NICU) admission was significantly higher in severe pre-eclampsia and non-severe pre-eclampsia compared to the controls.

**Table 6:** Comparison of NICU admission among severe pre-eclampsia, non-severe pre-eclampsia, and control patients. NICU: neonatal intensive care unit

NICU admission	Severe pre-eclampsia (n = 60)	Non-severe pre-eclampsia (n = 60)	Control (n = 60)	P-value
No	32 (53.33%)	32 (53.33%)	51 (85%)	0.0002 (significant)
Yes	28 (46.67%)	28 (46.67%)	9 (15%)	
Total	60 (100%)	60 (100%)	60 (100%)	

Both salivary and serum uric acid levels were significantly associated with maternal complications; however, there was no significant correlation between maternal complications and uric acid levels in the control group, as shown in Table 7.

**Table 7:** Association of uric acid with maternal complications in severe pre-eclampsia, non-severe pre-eclampsia, and control patients.

	Uric acid (mg/dL)	No complications	Complications present	Total	P-value
Severe pre-eclampsia	Salivary uric acid (mg/dL)				
	Mean ± SD	6.21 ± 0.3	6.84 ± 0.46	6.72 ± 0.49	0.0003 (significant)
	Range	5.82–6.54	5.9–7.6	5.82–7.6	
	Serum uric acid (mg/dL)				
	Mean ± SD	7.24 ± 0.27	8.33 ± 0.84	8.13 ± 0.87	0.0001 (significant)
	Range	6.81–7.6	6.2–9.41	6.2–9.41	
Non-severe pre-eclampsia	Salivary uric acid (mg/dL)				
	Mean ± SD	4.51 ± 0.92	5.04 ± 0.89	4.75 ± 0.94	0.006 (significant)
	Range	3.06–6.8	3.12–6.7	3.06–6.8	
	Serum uric acid (mg/dL)				
	Mean ± SD	5.95 ± 0.68	6.58 ± 0.71	6.23 ± 0.76	0.0007 (significant)
	Range	5.02–7.49	5.08–7.49	5.02–7.49	
Control	Salivary uric acid (mg/dL)				
	Mean ± SD	3.1 ± 0.39	3.55 ± 0.72	3.13 ± 0.43	0.105 (not significant)
	Range	2.09–4.6	3.01–4.6	2.09–4.6	
	Serum uric acid (mg/dL)				
	Mean ± SD	3.84 ± 0.44	4 ± 0.77	3.85 ± 0.46	0.755 (not significant)
	Range	2.99–5	3.12–5	2.99–5	

**Figure 3:**
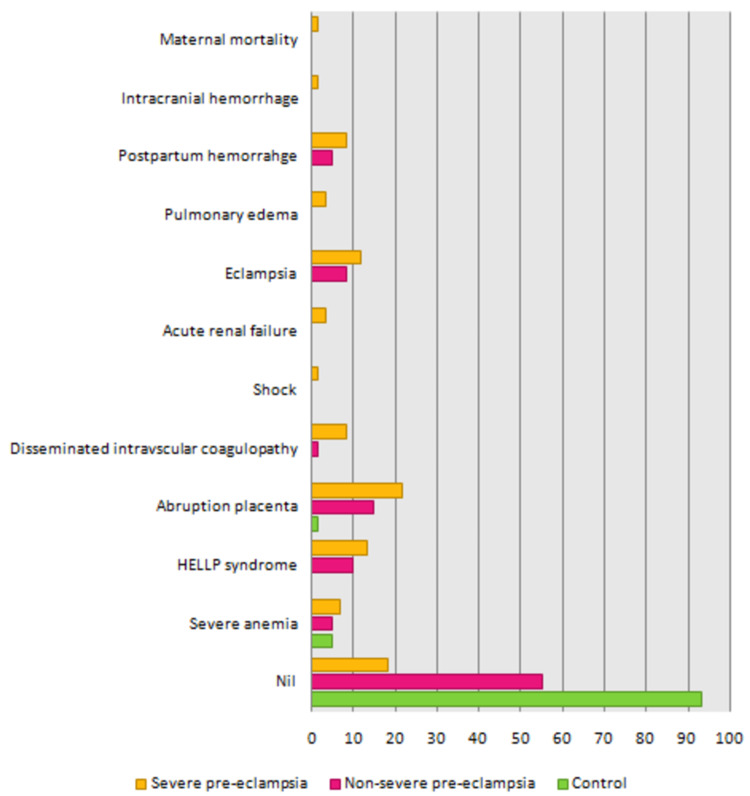
Comparison of maternal complications between severe pre-eclampsia, non-severe pre-eclampsia, and control. HELLP: hemolysis, elevated liver enzymes, low platelet count

As shown in Table 8 and Figure 3, patients without maternal complications were significantly higher in control (93.33%) compared to severe pre-eclampsia (18.33%) and non-severe pre-eclampsia (55%) (p-value < 0.0001). Severe pre-eclampsia had a significantly lower proportion of patients without maternal complications compared to control and non-severe pre-eclampsia patients.

**Table 8:** Comparison of maternal complications between severe pre-eclampsia, non-severe pre-eclampsia, and control patients. HELLP: hemolysis, elevated liver enzymes, low platelet count

Maternal complications	Severe pre-eclampsia (n = 60)	Non-severe pre-eclampsia (n = 60)	Control (n = 60)	Total	P-value
No maternal complications	11 (18.33%)	33 (55%)	56 (93.33%)	100 (55.56%)	<0.0001 (significant)
Severe anemia	4 (6.67%)	3 (5%)	3 (5%)	10 (5.56%)	1 (not Significant)
HELLP syndrome	8 (13.33%)	6 (10%)	0 (0%)	14 (7.78%)	0.007 (significant)
Placental abruption	13 (21.67%)	9 (15%)	1 (1.67%)	23 (12.78%)	0.004 (significant)
Disseminated intravascular coagulopathy	5 (8.33%)	1 (1.67%)	0 (0%)	6 (3.33%)	0.049 (significant)
Shock	1 (1.67%)	0 (0%)	0 (0%)	1 (0.56%)	1 (not significant)
Acute renal failure	2 (3.33%)	0 (0%)	0 (0%)	2 (1.11%)	0.33 (not significant)
Eclampsia	7 (11.67%)	5 (8.33%)	0 (0%)	12 (6.67%)	0.021 (significant)
Pulmonary edema	2 (3.33%)	0 (0%)	0 (0%)	2 (1.11%)	0.33 (not significant)
Postpartum hemorrhage	5 (8.33%)	3 (5%)	0 (0%)	8 (4.44%)	0.1 (not significant)
Intracranial hemorrhage	1 (1.67%)	0 (0%)	0 (0%)	1 (0.56%)	1 (not significant)
Maternal mortality	1 (1.67%)	0 (0%)	0 (0%)	1 (0.56%)	1 (not significant)

**Figure 4:**
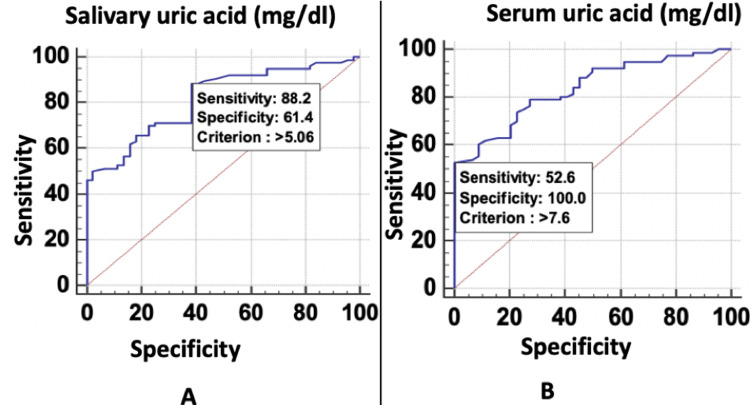
Receiver operating characteristic curve of (a) salivary and (b) serum uric acid for predicting complications in pre-eclampsia.

As shown in Figure 4, ROC curves above the diagonal line are considered to have a reasonable discriminating ability to predict complications in pre-eclampsia. Both parameters had significant discriminatory power to predict complications in pre-eclampsia. Discriminatory power of salivary uric acid (mg/dL) (area under the curve (AUC) = 0.815; 95% confidence interval (CI) = 0.734-0.880) and serum uric acid (mg/dL) (AUC = 0.835; 95% CI = 0.756-0.896) was excellent. Among all the parameters, serum uric acid (mg/dL) was the best predictor of complications in pre-eclampsia at a cut-off point of >7.6 with 83.50% chances of correctly predicting complications in pre-eclampsia.

Salivary uric acid (mg/dL) had a sensitivity of 88.16% followed by serum uric acid (mg/dL) with a sensitivity of 52.63%. In the prediction of complications in pre-eclampsia, serum uric acid (mg/dL) had the lowest sensitivity of 52.63%. On the other hand, serum uric acid (mg/dL) had a specificity of 100.00% followed by salivary uric acid (mg/dL) (61.36%). In the prediction of complications in pre-eclampsia, salivary uric acid (mg/dL) had the lowest specificity of 61.36%. The highest positive predictive value was found in serum uric acid (mg/dL) (100.00%) and the highest negative predictive value was found in salivary uric acid (mg/dL) (75.00%). Because there is always a trade-off between sensitivity and specificity (any increase in sensitivity will be accompanied by a decrease in specificity), we chose that variable as best in which combination of sensitivity and specificity gives the maximum predictive value, so overall serum uric acid (mg/dL) was the best predictor of complications in pre-eclampsia

**Figure 5:**
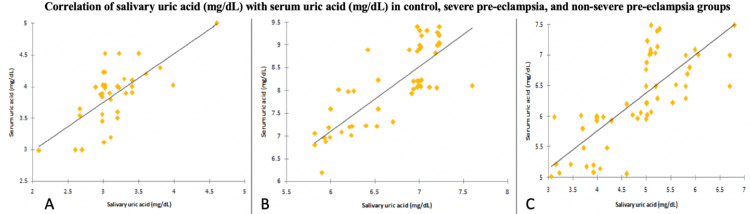
Correlation of salivary uric acid (mg/dL) with serum uric acid (mg/dL) in (a) control, (b) severe pre-eclampsia, and (c) non-severe pre-eclampsia patients.

There was a significant correlation between salivary and serum uric acid levels in all three groups with a p-value of less than 0.0001, as shown in Table 9 and Figure 5.

**Table 9:** Correlation of salivary uric acid (mg/dL) and serum uric acid (mg/dL).

Salivary uric acid (mg/dL) and serum uric acid (mg/dL)	Control	Severe pre-eclampsia	Non-severe pre-eclampsia	Pre-eclampsia
Correlation coefficient	0.598	0.827	0.818	0.920
P-value	<0.0001 (significant)	<0.0001 (significant)	<0.0001 (significant)	<0.0001 (significant)

## Discussion

HDP are an important cause of peripartum mortality which need to be addressed, especially in developing nations, such as India, where there is a lack of awareness and scarcity of health resources in remote areas. Various studies have been conducted in the past assessing serum uric acid levels; however, there is a paucity of data on salivary uric acid levels in patients with eclampsia.

Singh et al. (2019) [33] observed a statistically significant correlation (r^2^ = 0.690, p = 0.001) between saliva uric acid and serum uric acid of women diagnosed with pre-eclampsia. This study also observed a similar correlation between serum uric acid and salivary uric acid among all the three groups, i.e., severe pre-eclampsia (r = 0.827, p < 0.0001), non-severe pre-eclampsia (r = 0.818, p < 0.001), and pre-eclampsia (r = 0.920, p < 0.001). In the study by Gautam et al. (2020) [34], a linear correlation was observed between serum and salivary values of uric acid (r = 0.334, p = 0.006). Riis et al. (2018) [35] found robust positive saliva serum correlation for uric acid.

In this study, a statistically significant difference was found in the mean serum uric acid levels among cases of severe pre-eclampsia who underwent normal vaginal delivery or cesarean section. The mean serum uric acid level was significantly higher in cases where LSCS was performed. The results concluded that serum uric acid can predict the mode of delivery (normal vaginal or cesarean) among cases of severe pre-eclampsia. These results were corroborated by another study by Kumar et al. (2019) [36], where a significant difference was observed among cases who underwent normal vaginal delivery and cesarean section. In the study by Padma et al. (2015) [37], high serum uric acid levels were associated with delivery by cesarean section.

In this study, the proportion of patients with NICU admission was significantly higher in severe pre-eclampsia and non-severe pre-eclampsia compared to control (46.67%, 46.67%, and 15%, respectively). Similar results were observed in the study conducted by Sachan et al. (2013) [38], where 25.9% of the neonates of mothers with severe pre-eclampsia required NICU admissions compared to the controls where only 6.5% NICU admissions occurred. Similar results were observed in the study conducted by Wolde et al. (2011) [39], where 15.3% of the neonates born to mothers with severe pre-eclampsia were admitted to NICU in comparison to 0.9% of the neonates born to mothers with mild pre-eclampsia.

In the present study, patients without maternal complications were significantly higher in control compared to severe pre-eclampsia and non-severe pre-eclampsia. Most maternal complications were found in the severe pre-eclampsia group, followed by the non-severe pre-eclampsia and control groups. Women faced abruptio placenta (21.67%), HELLP syndrome (13.33%), disseminated intravascular coagulopathy (8.33%), and eclampsia (11.6%) significantly higher if they were suffering from severe pre-eclampsia in comparison to the controls. Similar findings were observed in the study conducted by Sachan et al. (2013) [38], where abruption was faced by 25% and eclampsia was faced by 27.27% of the women categorized under severe pre-eclampsia. Similar results were observed in the study by Irene et al. (2021) [40], where complete and incomplete HELLP syndrome (20.8% and 11.3%) was faced by women having eclampsia. Another study by Belay et al. (2018) [41] showed similar results with maternal complications (30.7) including placental abruption (5%), HELLP syndrome (2.4%), and disseminated intravascular coagulopathy (1.22%). Similar findings were also observed in the study conducted by Saadat et al. (2015) [42], where placental abruption was faced by 8.8% of women in the pre-eclampsia group.

In the study by Sachan et al. (2013) [38], the majority of control patients (87.10%) belonged to middle socioeconomic status while 67.69% of mild pre-eclampsia and 71.88% of the severe pre-eclampsia cases belonged to the middle socioeconomic class. A significant association was observed between socioeconomic status and the different groups. The present study also states a significant association between the socioeconomic status of patients in the three groups. The majority of the patients in the control group belonged to the upper class (20%) and upper-middle class (35%), whereas the majority of the patients in the severe pre-eclampsia group belonged to the lower class (36.67%) and lower middle class (30.67%), and patients from the non-severe pre-eclampsia group belonged predominantly to the lower middle class (33.33%) and middle class (25%). This distribution of socioeconomic status was statistically significant.

Low socioeconomic and educational status predisposes women to poor living conditions, which, in turn, make women susceptible to high-risk conditions in pregnancy. Education has been closely linked to the health status and the prenatal care received by women, especially in rural settings. Due to lack of education, women do not seek health care, so many cases of HDP remain uncared and untreated during pregnancy for early diagnosis. Moreover, some researchers have suggested that women with lower socioeconomic status have more stress so there are more chances of developing HDP. In such a setting of low socioeconomic strata, salivary uric acid can act as a cost-effective wonder in HDP and potentially in other pathological conditions where serum uric acid levels are raised [43-46].

Limitations

The main limitation of this study was the small sample size and that it was conducted in a single center. Therefore, further studies with a larger sample size are required to establish salivary uric acid as an important tool in managing pre-eclampsia.

## Conclusions

Salivary uric acid is significantly related to maternal complications and NICU admission in patients who have pre-eclampsia. It was also significantly related to serum uric acid levels, thus providing a good tool that is both time and cost-effective to provide an insight into serum uric levels and prognoses in cases of pre-eclampsia. Salivary and serum uric acid levels were also found to be significantly raised in patients undergoing cesarean section with significantly higher levels of uric acid levels in serum and saliva in patients undergoing emergency cesarean section in patients of severe and non-severe pre-eclampsia. Although the specificity of salivary uric acid was less than serum uric acid levels, it had high sensitivity which may aid in establishing salivary uric acid as a screening tool for diagnosing pre-eclampsia, predicting obstetric complications and neonatal intensive care unit admission, especially in rural centers. Further studies can establish salivary uric levels as a useful tool to be used in rural obstetric HDUs to stratify and manage pre-eclamptic patients based on risk assessment.
